# The Effect of OSM on MC3T3-E1 Osteoblastic Cells in Simulated Microgravity with Radiation

**DOI:** 10.1371/journal.pone.0127230

**Published:** 2015-06-01

**Authors:** Jake Goyden, Ken Tawara, Danielle Hedeen, Jeffrey S. Willey, Julia Thom Oxford, Cheryl L. Jorcyk

**Affiliations:** 1 Department of Biological Sciences, Boise State University, 1910 University Drive, Boise, Idaho 83725, United States of America; 2 Department of Radiation Oncology, and the Comprehensive Cancer Center, Wake Forest School of Medicine, 1 Medical Center Blvd, Winston-Salem, North Carolina, 27157, United States of America; 3 Biomolecular Research Center, Boise State University 1910 University Drive, Boise, Idaho 83725, United States of America; University of Texas Southwestern Medical Center, UNITED STATES

## Abstract

Bone deterioration is a challenge in long-term spaceflight with significant connections to patients experiencing disuse bone loss. Prolonged unloading and radiation exposure, defining characteristics of space travel, have both been associated with changes in inflammatory signaling via IL-6 class cytokines in bone. While there is also evidence for perturbed IL-6 class signaling in spaceflight, there has been scant examination of the connections between microgravity, radiation, and inflammatory stimuli in bone. Our lab and others have shown that the IL-6 class cytokine oncostatin M (OSM) is an important regulator of bone remodeling. We hypothesize that simulated microgravity alters osteoblast OSM signaling, contributing to the decoupling of osteolysis and osteogenesis in bone homeostasis. To test this hypothesis, we induced OSM signaling in murine MC3T3-E1 pre-osteoblast cells cultured in modeled microgravity using a rotating wall vessel bioreactor with and without exposure to radiation typical of a solar particle event. We measured effects on inflammatory signaling, osteoblast activity, and mineralization. Results indicated time dependent interactions among all conditions in the regulation of IL-6 production. Furthermore, OSM induced the transcription of OSM receptor ß, IL 6 receptor α subunits, collagen α1(I), osteocalcin, sclerostin, RANKL, and osteoprotegerin. Measurements of osteoid mineralization suggest that the spatial organization of the osteoblast environment is an important consideration in understanding bone formation. Taken together, these results support a role for altered OSM signaling in the mechanism of microgravity-induced bone loss.

## Introduction

Deteriorating bone health is a substantial barrier to human exploration deeper into the solar system [1]. Each month in space, astronauts lose approximately 1% of the mineral density in their weight-bearing bones [2–5]. Travel to Mars or near-Earth asteroids could last two years or more, greatly extending the astronauts’ exposure to the risks of spaceflight most relevant to bone health: microgravity and ionizing radiation. Galactic cosmic rays (GCR) and solar particle events (SPE) become important sources of radiation to astronauts, where they may be exposed to more than 2 Gy of radiation [6,7]. These doses are comparable to those used in cancer radiotherapy to kill tumor cells. There are also recognized clinical parallels between spaceflight bone loss and disuse osteoporosis, a common complication of inactivity from bed rest, immobilization, or sedentary lifestyle{FormattingCitation}. To date, efforts to counter microgravity-mediated bone demineralization have focused on resistive exercise and nutrition with limited success [2–4,11]. Taken together, these facts imply that pharmacological countermeasures to bone loss will likely be required for exploration deeper into the solar system; however, an increased understanding of relevant molecular pathways is needed to identify possible targets for intervention.

The bone remodeling cycle is a multicellular process where bone is constantly broken down and replaced [12–16]. Bone is calcified extracellular matrix (ECM) produced by osteoblasts, whose differentiation from mesenchymal stem cells (MSC) is determined primarily by the transcription factor RUNX2 [17]. After proliferation and additional maturation that is marked by the transcription factor osterix[18], the osteoblast will produce osteoid, the organic component of bone ECM that is primarily composed of type I collagen. Additional factors such as osteocalcin regulate the mechanical and chemical properties of the bone [19]. As the ECM matures, osteoblasts become encased and terminally differentiate into osteocytes [20]. An important function of the osteocyte is to limit the excessive formation of bone, largely through the production of sclerostin [21,22]. The osteoblast lineage cells also initiate the bone remodeling cycle by recruiting osteoclasts, which resorb damaged bone [23,24]. Osteoclasts differentiate from myeloid precursors, primarily under the influence of macrophage colony stimulating factor (MCSF) and receptor activator of nuclear factor κB ligand (RANKL) that are produced by the osteoblast lineage [25]. The bone remodeling cycle is balanced by regulated activity of osteoblasts and osteoclasts to adapt to changing mechanical and gravitational demands on the bone [13,23].

Microgravity leads to a dysregulation of the activity between osteoblasts and osteoclasts, leading to bone degradation [26–28]. Very little is known about the combined effects of microgravity and radiation on bone, as the vast majority of publications using ground-based models examine only one factor in isolation [3,29,30]. One potential mechanism suggested for the disruption of bone homeostasis in spaceflight includes disregulation of inflammatory cytokines such as interleukin-6 (IL-6) [31–34], where ground-based experiments have shown increased activity of this inflammatory pathway [26,28,35]. Similar alterations in inflammatory signals are also seen in disuse osteoporosis [8–10] and radiation exposure [36–38].

The gp130 family of cytokines, which include IL-6, leukemia inhibitory factor (LIF), and oncostatin M (OSM), are an important group of factors with broad effects in human bone health and disease [39]. IL-6 signals through a complex comprised of the IL-6 receptor α subunit (IL-6Rα) and a homodimer of GP130. LIF signals through a complex of GP130 and the LIF receptor α subunit (LIFRα) [39]. OSM signals through either the LIFRα —GP130 dimer or a dimer of GP130 and the OSM receptor ß subunit (OSMRß). All of the major cell types in bone express some combination of GP130 cytokines and their receptors [39], and OSM has been shown to induce IL-6 secretion by osteoblasts [40]. IL-6 has been shown to promote both mineralization and demineralization by stimulating osteoblast differentiation [41] and activation of osteoclasts, respectively [41,42,43]. Perception of OSM has been colored by the role of its better-known relative IL-6. Indeed, OSM has been shown in cell culture to promote osteoclastogenesis by inducing osteoblast RANKL expression and reducing expression of osteoprotegerin [44–47]. However, it has also been shown that OSM promotes differentiation and activation of osteoblasts, mineral formation, and represses the expression of sclerostin [40,48–51]. *In vivo* data are complicated and suggest that some effects are species-specific [52]. Clearly, many details of the action of OSM in bone remain to be illuminated, as it is unclear whether OSM causes net bone formation or destruction.

OSM is known to act directly on osteoblasts and indirectly on osteoclasts, and changes in OSM action could account for many of the effects of microgravity on bone. Defining a connection amongst OSM, osteoblast activity, and radiation response would contribute to our understanding of OSM in bone. In this study, we examined the actions of OSM on gp130 signaling and osteoblast activity in a model of simulated microgravity with and without radiation. Our model system used the MC3T3-E1 mouse pre-osteoblast cell line, which has been shown to recapitulate important actions of OSM seen in primary cells [49,53,54]. Our results support the hypothesis that simulated microgravity conditions along with radiation alter the action of OSM signaling in osteoblasts. Specifically, OSM and microgravity both increased the production of the inflammatory cytokine IL-6 in the MC3T3-E1 osteoblastic cell lines. Furthermore, radiation in combination with microgravity also increased IL-6 production in the cell lines.

## Materials and Methods

### Cell Culture

The MC3T3-E1 subclone 4 mouse pre-osteoblast cell line [55] and the UMR-106 rat osteosarcoma cell line were obtained directly from the American Type Culture Collection (Rockville, MD). MC3T3-E1 cells were maintained in MEM (Life Technologies, Grand Island, NY) supplemented to make α-MEM without ascorbic acid. All supplement components were from Sigma-Aldrich (St. Louis, MO). UMR-106 were maintained in DMEM (Hyclone, Logan, UT). All culture media were supplemented with 10% fetal bovine serum and 100 U/mL each of penicillin and streptomycin. These supplements were obtained from Hyclone (Logan, UT). Cells were maintained at 37°C, 5% carbon dioxide, and 95% humidity.

### Microcarrier Culture

Cytopore 2 macroporous cellulose microcarriers were purchased from GE Healthcare (Pittsburgh, PA). Microcarriers were hydrated in phosphate buffered saline (PBS) at 20 mg/mL and autoclaved for 20 min at 121°C. Microcarriers were rinsed twice in sterile PBS then transferred to culture medium for at least 16 h prior to seeding cells.

To seed the microcarriers, MC3T3-E1 cells were grown to confluence, trypsinized, and suspended in culture medium with microcarriers in a tissue culture flask. Concentration of cells during seeding was 50,000 cells/mL and 5 mg microcarriers/mL. The mixture was agitated by gentle pipetting every 20 min for 3 h and then adjusted to 2 mg/mL. After seeding microcarriers were incubated as above. Microcarriers were agitated each morning and afternoon. Approximately one-half the medium was replaced every three to four days. Experiments were initiated on the seventh day after seeding.

### Osteogenic Differentiation and Cytokine Stimulation

To induce osteogenic differentiation, MC3T3-E1 cells were transferred to standard α-MEM (Life Technologies) supplemented with an additional 50 μg/mL ascorbic acid and 5 mM phosphate buffer at pH 7.4. Mineralization medium was also supplemented as above. Cells cultured on microcarriers had supernatant medium removed and replaced with differentiation medium with mixing 3 times. To induce OSM signaling, culture medium was supplemented with 25 ng/mL recombinant mouse OSM (R&D Systems).

### Rotary Cell Culture System (RCCS)

Cells were cultured on Cytopore-2 cellulose microcarriers for use in the NASA-developed Rotary Cell Culture System (RCCS). This rotating wall vessel (RWV) type bioreactor models microgravity through gravitational vector averaging and low shear stress [56,57]. The Rotary Cell Culture System (RCCS) and 10-mL high aspect ratio vessels (HARVs) were purchased from Synthecon (Houston, TX). The HARV rotates around a horizontal axis so that medium and microcarriers undergo solid body rotation, which averages the gravitational vector experienced by cells to near zero. For Cytopore 2 microcarriers, a rotational speed of 18 rpm was found to be optimal by visual inspection. Oxygenation occurs through a gas-permeable membrane, preventing the formation of bubbles and ensuring smooth rotation for the microcarriers.

Modeled microgravity experiments lasted 7 days. At 12 h, 48 h, 96 h, and 7 days, samples were collected for analysis. After ensuring uniform suspension of the microcarriers, a portion of the medium was retained so that the microcarriers would be evenly divided among the samples. The remaining microcarriers were returned to the HARV along with fresh medium matching the experimental condition. As controls at normal gravity, microcarriers were cultured in tissue culture flasks at identical concentrations and volumes to RCCS in the incubator at 37°C and 5% CO_2_, as described in previous studies involving microgravity [58–60]. Subsequent samples were collected by the same procedure.

### Radiation

MC3T3-E1 cells were prepared on Cytopore 2 microcarriers as above. Approximately 18 h prior to irradiation, cells were transferred to a sterile 50 ml conical tube at a concentration of 2 mg Cytopore per mL. This concentration provided excess culture medium. The tubes were then packed in an insulated, pre-warmed box with a 2 L bottle of water at 37° C and shipped overnight to our collaborator Dr. Jeffrey Willey. Tubes were exposed to 1 Gy at 364 rad/s from a ^137^Cs source. Tubes were repacked and return shipped overnight. Controls were subjected to a sham irradiation procedure. Upon return, cells were immediately transferred to mineralization medium and modeled microgravity or control conditions. Samples were collected as described above at 12 h, 48 h, 96 h, and 7 days.

### Semi-Quantitative Reverse Transcription-Polymerase Chain Reaction (RT-PCR)

Microcarriers were allowed to settle in a conical tube and the supernatant medium was aspirated. RNA was extracted using 1 mL RNA-STAT 60 (Tel-test Friendswood, TX) for each 1–2 mg microcarriers. After a 20-min incubation, chloroform (200 μL/1 mL of RNA-STAT 60) was added followed by vortexing for 10 seconds and centrifugation at 12,000 rpm. The upper aqueous layer was transferred to a new tube containing isopropanol (0.5 mL/1 mL of RNA STAT60), followed by vortexing for 10 seconds and incubation on ice for 15 min. The mixture was centrifuged at 12,000 rpm and the supernatant discarded. The pellet was washed with 1 mL of 75% ethanol and centrifuged at 12,000 rpm, and then the supernatant discarded. The RNA was allowed to air-dry in a sterile environment and resuspended with nuclease-free water.

cDNA was generated from this RNA using a commercially available reverse transcriptase kit (Applied Biosystems) per manufacturer instructions. The cDNA generated from the reverse transcription reaction was used in a 25 **μ**L PCR reaction containing 2.5 **μ**L of 10x PCR buffer, 2.5mM dNTPs, 10 mM primers, 5U GoTaq polymerase, (Promega, Madison, WI) and 2 **μ**L cDNA. Amplifications were carried out as follows: initial denaturation at 95°C for 2 min, followed by the indicated number of cycles of 95°C for 1 min, annealing temperature for 1 min, 72°C for 1 min, then a final extension of 72°C for 10 min. Primer pairs and reaction conditions for each target are provided in Table 1. The PCR products were electrophoresed on a 1% Tris-Agarose gel containing 0.5 **μ**g/mL of ethidium bromide at 80 volts for 45 min. The gels were imaged using a Kodak Image station and exposed for 10 seconds. Band densities for all semi-quantitative PCR were calculated using the ImageJ software (NIH) and normalized to GAPDH. The sample size for OSM-treated cells with or without microgravity was four, and the sample size for studies including radiation was three.

**Table 1:** PCR Primers and Reaction Conditions.

Target	Fwd. Primer Rev. Primer	Prod. Size	Temp. (°C)	Cycles	cDNA dilution
GAPDH	ATCACTGCCACCCAGAAGAC GGTCCTCAGTGTAGCCCAAG	202	57	30	1:10
OSM	AGCAAGCCTCACTTCCTGAG GTGGGCTCAGGTATCTCCAG	200	60	35	1:01
OSMRβ	TAGACTGAACATATCCAACACCA TCCATGGATTGGCTCATCTGGCA	349	60	30	1:01
LIF	CAGACAGACAGGTAGCATAAAG GACACAGAGACAGACAGAGA	487	60	35	1:01
LIFRα	GAAAACTGTAAGGCGCTACA CCAAGTGTTTACATTGGC	483	52	35	1:01
IL-6	CCTCTGGTCTTCTGGAGTACCAT GGCATAACGCACTAGGTTTGCCG	307	55	30	1:10
IL-6Rα	CCAGGTGCCCTGTCAGTATT CCGTGAACTCCTTTGACCAT	317	60	35	1:01
MCSF	CGACTTCCCGTAAAGGCATAAA CAAGGAACACAGCCCAAAGA	530	60	30	1:01
RANKL	GAGAGGTATTCCGATGCTTATG GGTGACCAACATCCTACTTATT	577	60	35	1:01
Osteoprotegerin	AGAGTGAGGCAGGCTATT AGTAGTTTCTTCTGGTGCTATG	511	60	35	1:01
RUNX2	CCCTTCCTCTTCCCTTATCTCT GTGCTTCTGCTACCACTCTAAC	509	60	35	1:01
Osterix	CTGCTTGAGGAAGAAGCTCACTA GGGGAGCAAAGTCAGATGGG	490	60	35	1:01
Collagen α1(I)	AACAAGGTGACAGAGGCATAAA GCTGCGGATGTTCTCAATCT	440	60	30	1:10
Osteocalcin	GACCATCTTTCTGCTCACTC TTGCACTTCCTCATCTGAAC	425	60	35	1:01
Sclerostin	TTCCACCCAAATGTAAAGCCTGCG ATTTCTGGCCCTTCCACCATCTCT	366	60	35	1:01

### Enzyme-Linked Immunosorbent Assay

To measure secreted IL-6, conditioned medium was collected from each sample collected above. IL-6 was quantified with the mouse IL-6 DuoSet kit from R&D Systems according to the manufacturer’s instructions. Plates were washed with PBS at pH 7.4 containing 0.05% Tween-20 (PBS-T) and blocked using PBS containing 1% IgG-free BSA (Jackson Immunological West Grove, PA). The substrate used was Thermo Pierce (Rockford, IL) 1-Step Ultra TMB. A seven point standard curve was prepared by serial dilution of the included 1000 pg/mL standard. Sample concentrations were interpolated from a 4 parameter logistic fit of the standards. All samples and standards were assayed in duplicate. All ELISA analyses had a sample size of three.

### Alizarin Red Staining

For monolayer experiments, MC3T3-E1 cells were cultured in mineralization medium for 6 or 14 days in 12-well plate. For microcarrier experiments, a 2 mg sample was retained from each RCCS experiment and cultured for an additional week in experimental conditions. Medium was aspirated and the cells were washed once with PBS and then fixed in 10% formalin for 15 min. Formalin was aspirated and the cells were washed three times with deionized water. Four hundred μL of 40 mM alizarin red (Millipore, Billerica, MA) was added to each sample. After a 20-min incubation at room temperature, the stain was aspirated and the cells were washed four times with deionized water.

For extraction and spectrophotometric quantification, 400 **μ**L 10% acetic acid was added to each well. The matrix was disrupted with a pipette tip in monolayer or trituration in microcarrier experiments. The sample was then transferred to (or retained in) a microcentrifuge tube and incubated for 30 min at 85°C. Tubes were then transferred to ice for 5 min and then centrifuged 20 min at 16,000 x g. Ten standards were prepared by serial dilution from 4 mM alizarin red. Samples and standards were adjusted to pH 4.2 with 10% ammonium hydroxide. One hundred **μ**L of each sample and standard was transferred in duplicate to a 96-well plate and the absorbance read at 405 nm. Sample concentration was calculated by comparison to a linear least-squares best fit of the standards.

For quantification by confocal microscopy and densitometry, a microscope slide of the sample was prepared after washing but before the extraction procedure. The sample was imaged on a Zeiss LSM 510 Meta system combined with the Zeiss Axiovert Observer Z1 inverted microscope and ZEN 2009 imaging software (Carl Zeiss, Inc., Thornwood, NY). Excitation was at 540 nm and emission was measured at 580 nm. A sample of at least 20 microcarriers was imaged under identical settings with intensity at 580 nm saved as 8-bit grayscale. Using the ImageJ software (NIH), threshold was applied at 30/255 to eliminate background and the integrated intensity of each image was calculated. Integrated intensity per bead was calculated and used to represent alizarin red staining.

### Statistics

RCCS experiments were analyzed using three-way analysis of variance (ANOVA) with repeated measures of each combination of the RCCS and OSM induction (corresponding to a culture vessel) at each level of time. The RCCS with radiation experiments were analyzed using four-way ANOVA with repeated measures of each combination of radiation, RCCS, and OSM induction (corresponding to a culture vessel) at each level of time. Multiple comparisons were conducted with Tukey's HSD test *post hoc*. Each response variable was treated separately. For all comparisons, α = 0.05. In figures, bars and asterisks (*) indicate p < 0.05 for the indicated main effect. Any interactions identified in the test had a p < 0.05. Calculations were performed in the R statistical environment (R Project).

## Results

### OSM and RCCS synergistically induce IL-6 secretion

**Fig 1:**
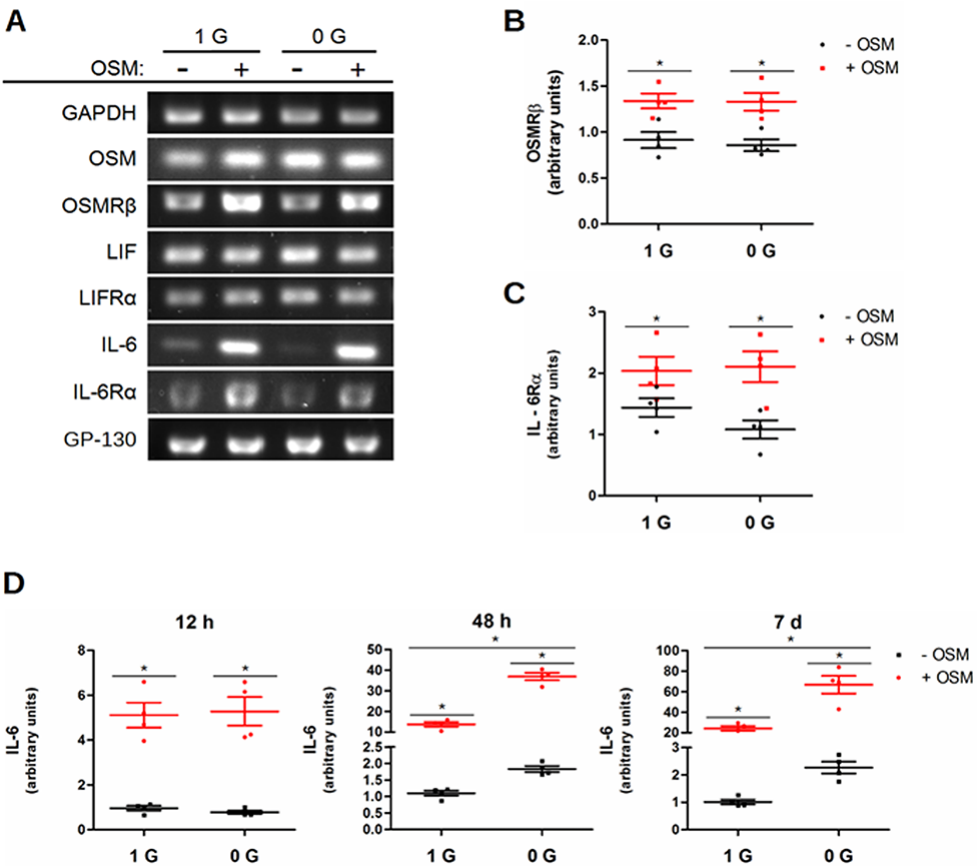
OSM and RCCS independently and synergistically induce the transcription of IL-6. MC3T3-E1 pre-osteoblasts were cultured with OSM supplementation and RCCS for 7 days. RNA was collected at 12 h, 48 h, and 7 d. Semi-quantitative RT-PCR was conducted for the cytokines and receptors most closely associated with OSM in osteoblasts, listed in (A) with representative images from 48 h, when effects were seen for the largest number of targets. (B-D) Scatter plots of the densitometry results showing the mean and standard error of the mean (SEM) for the targets with statistically significant regulation by OSM or RCCS (arbitrary units). OSM alone induced the (B) OSMRß and (C) IL-6Rα subunits, shown at 48 h, when the largest effect was seen. (D) IL-6 was induced independently by both OSM and RCCS. Induction by OSM alone progressed from approximately 5-fold at 12 h to 20 fold at 7 d. Induction by RCCS was first significant after 48 h and remained stable afterward at approximately 2-fold. The factors interacted significantly to amplify their independent effect, inducing IL-6 transcription approximately 35-fold at 48 h and 70-fold at 7 d.

To assess the action of osteoblast OSM signaling in the RCCS, we first examined the transcription of the cytokines and receptors most closely associated with OSM by semi-quantitative RT-PCR (Fig 1A). OSM supplementation was observed to induce the transcription of OSMRß (Fig 1B) and IL-6Rα (Fig 1C). Induction of the receptors reached its peak at 48 h and remained stable afterward. These effects of OSM have not been previously described in osteoblasts. RCCS had no statistically significant effect on these targets.

IL-6 was the most substantially affected of the examined transcripts (Fig 1D). Both OSM supplementation and RCCS independently induced IL-6 transcription, which is consistent with prior results [27,40]. Additionally, the combination of OSM supplementation and RCCS (hereafter OSM+RCCS) increased IL-6 transcription by more than twice what would be expected from even multiplicative combination of their individual effects, reaching 70-fold by 7 d. This synergistic effect has not previously been described. OSM's effect was seen as early as 12 h after treatment and increased throughout the 7 days. The independent effect of RCCS was not observed until 48 h and remained stable afterward. The synergistic effect from OSM+RCCS was detected from 48 h, along with the effect from RCCS alone. The amplification of IL-6 induction demonstrates for the first time that RCCS does alter the effect of OSM signaling.

**Fig 2:**
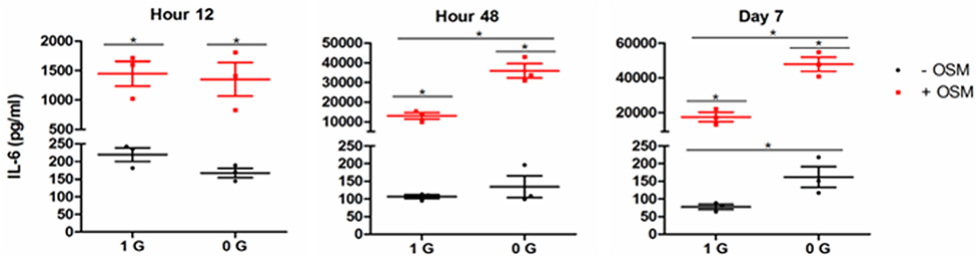
OSM and RCCS independently and synergistically induce secretion of IL-6. MC3T3-E1 pre-osteoblasts were cultured with OSM supplementation and RCCS for 7 d. IL-6 secretion was measured in conditioned medium by ELISA; scatter plots show mean and SEM. OSM treatment induces secretion of IL-6 at all time points with a 7-fold induction at 12 h increasing to 200-fold by 7 d. A 2-fold increase in IL-6 secretion by RCCS is significant at 7 d, but may be present earlier. The synergistic effect of OSM and RCCS on IL-6 secretion is first significant at 48 h at approximately 350-fold. It increases to approximately 500-fold at day 7.

To ensure that the effects of OSM signaling induction and RCCS extended to the secretion of the IL-6 protein, we tested the cell culture conditioned medium using ELISA (Fig 2). Again, both OSM supplementation and RCCS were shown to independently and synergistically induce osteoblast IL-6 production, with the effect of RCCS lagging OSM in both time and scale. OSM alone increased secretion by as much as 200-fold over control conditions, while the effects of RCCS on mRNA and secreted protein were proportional at 48 h. The fold increase in secreted protein was much larger than the transcriptional change. This may reflect the accumulation of protein in the culture medium as IL-6 was produced at increasing rates, as well as its sequestration after binding to extracellular matrix proteins secreted by the osteoblasts [61]. It is also possible the at post-transcriptional regulatory effects account for difference. Regardless, the interaction of RCCS and OSM is confirmed at the protein level. This supports the hypothesis that microgravity alters OSM signaling in the osteoblast.

### Radiation limits the effect of OSM induction on IL-6 secretion, but enhances the effect of RCCS

**Fig 3:**
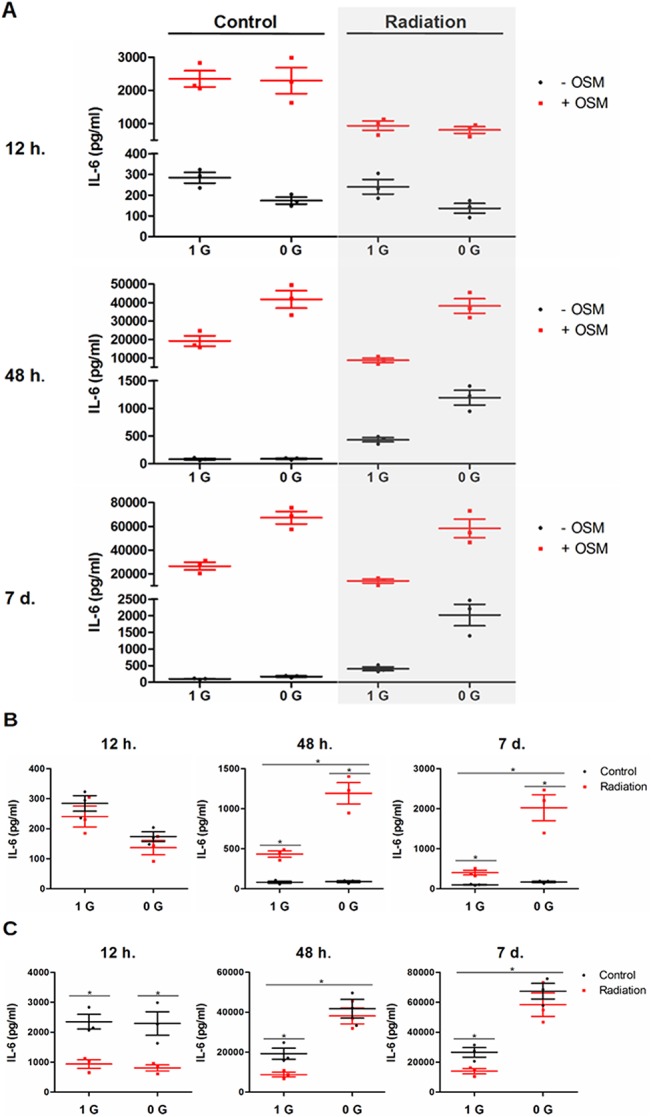
Radiation limits the effect of OSM induction on IL-6 secretion but enhances the effect of RCCS. MC3T3-E1 cells were cultured in a spaceflight model combining culture in the RCCS and radiation representative of a SPE (1 Gy at 364 rad/s from a ^137^Cs source). Osteoblast IL-6 secretion was measured by ELISA and are shown as scatter plots with mean and SEM. An overview of these data is shown in (A). The effects of OSM and RCCS on IL-6 secretion were unaffected by sham irradiation (A, left column). Under all conditions, OSM treatment induced IL-6 secretion by at least 10-fold. Consequently, radiation and RCCS effects are broken out into control (B) and OSM treated (C). In the absence of OSM treatment (B), irradiation alone increased IL-6 secretion relative to sham irradiation by approximately 5-fold from 48 h on. The combination of RCCS and radiation treatments (without OSM treatment, B) magnified the induction of IL-6 approximately 10-fold compared to RCCS treatment alone. With OSM treatment (C), irradiation without RCCS decreased IL-6 secretion at all time points. Irradiation had no effect on the synergistic increase in IL-6 secretion seen with combined OSM induction and RCCS. For clarity, statistical results are shown only in (B) and (C).

The action of OSM signaling on osteoblast inflammatory factors was next examined in the context of a more complete model of spaceflight including both simulated microgravity and radiation typical of a solar particle event (SPE). Statistically significant interactions (p < 0.01) between OSM signaling and these spaceflight conditions were again observed in the regulation of IL-6 production (Fig 3). Radiation increased IL-6 expression but had no significant additional effect on any other targets assessed by RT-PCR, including OSM, OSMRß, LIF, LIFRß, IL-6Rα, MCSF, RANKL, osteoprotegerin, RUNX2, osterix, collagen α1(I), osteocalcin, and sclerostin (data not shown). The changes in IL-6 mRNA measured by RT-PCR were entirely reflected in the measurements of secreted IL-6 (Fig 3). A general increase in the concentration of IL-6 compared to the experiments without radiation is attributed to the increase in Cytopore microcarrier concentration from 4 mg/mL to 4.5 mg/mL, increasing the number of cells, and the fewer time points at which samples were collected, decreasing the dilution of the conditioned medium with fresh medium. Both changes were made to provide sufficient cell numbers for the additional experimental factor. Sham irradiation control recapitulated the effects of the RCCS-only model with its substantial synergistic increase in secretion (Fig 3A, left column), validating the radiation procedure. As in the experiments without radiation, OSM increased IL-6 secretion by at least 10-fold under all conditions. Consequently, to facilitate comparison of other conditions, the results of the experiments with radiation are separated into panels showing results without-OSM (Fig 3B) and with-OSM (Fig 3C).

Intriguingly, the effects of radiation in combination with OSM supplementation were opposite of the effects of radiation alone or radiation with RCCS. Radiation alone increased IL-6 secretion relative to the control at 48 h and 7 d by approximately 5-fold, confirming a response recently reported for the first time in osteoblasts (Fig 3B) [37]. Radiation in combination with RCCS increased the secretion of IL-6 relative to either factor alone. The scale of this increase compared to multiplicative combination of the factors was approximately two-fold, comparable to the synergistic effect seen from OSM+RCCS in absence of radiation. Contrary to these increases in IL-6 secretion, irradiation decreased the effect of OSM supplementation at all time points by a substantial margin, approximately 50% (Fig 3C). Finally, radiation did not change the induction of IL-6 by OSM+RCCS except at 12 h, when all conditions respond as if RCCS were not present. Considered together, these complicated interactions again support the action of microgravity conditions on OSM signaling.

### OSM counteracts the effect of RCCS on the RANKL: osteoprotegerin ratio

**Fig 4:**
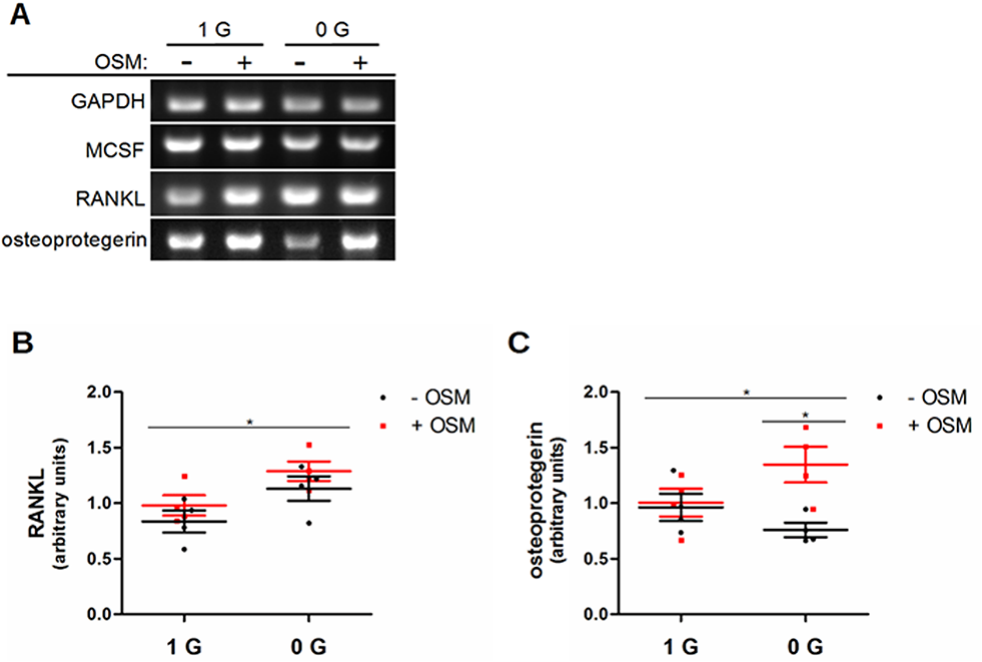
OSM counteracts the effect of RCCS on the RANKL: osteoprotegerin ratio. Semi-quantitative RT-PCR was used to examine the interaction of OSM and modeled microgravity on the osteoblast transcription of MCSF, RANKL, and osteoprotegerin. (A) Representative images from samples collected after 48 h, when the most significant effects were seen for RANKL and osteoprotegerin. No changes were detected for MCSF or with radiation for any of these targets. Significant effects when quantified by densitometry are shown as scatter plots with mean and SEM for RANKL (B) and osteoprotegerin (C). RANKL transcription increased under RCCS (B), without an associated increase in osteoprotegerin (C). The combination of OSM and RCCS, however, increased osteoprotegerin proportionally to the increase in RANKL.

It has been observed that IL-6 functions primarily in bone to magnify osteoclast recruitment and activity [42,43]. OSM is also known to enhance RANKL expression and osteoclast activation [44,46,47]. To determine if microgravity conditions also interact with these actions of OSM, we measured the transcription of the osteoblast-produced factors most important for osteoclastogenesis: MCSF, RANKL, and osteoprotegerin (Fig 4A). We found that RANKL (Fig 4B) was upregulated by RCCS. RCCS alone also decreased the transcription of osteoprotegerin (Fig 4C), so that the ratio of RANKL to its decoy receptor would be increased, which favors increased osteoclastogenesis. In these experiments, OSM did not exert a statistically significant effect on RANKL expression, but there was a clear interaction between OSM signaling induction and RCCS. When the two were present in combination, osteoprotegerin mRNA levels increased proportionally to the increase seen in RANKL, so that in this case the RANKL: OPG ratio would be preserved. The implication is that the osteoblast recruitment of osteoclasts in microgravity may depend on the absence of OSM signaling.

### OSM and RCCS have independent and opposing effects on osteoblast activity

**Fig 5:**
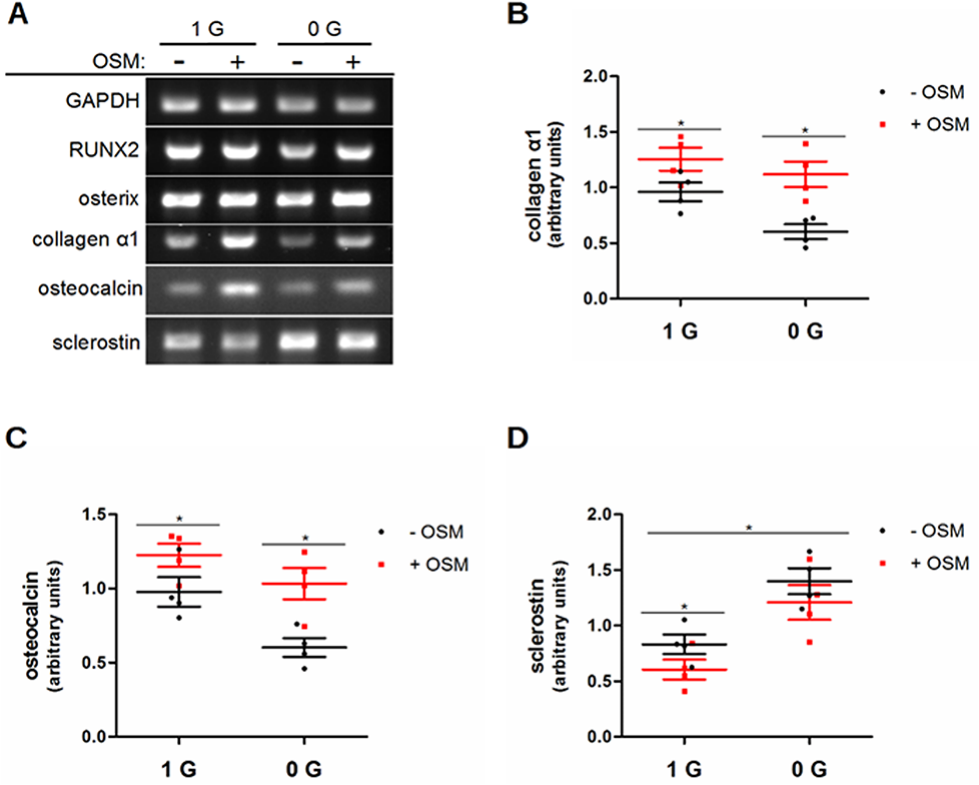
OSM and RCCS have independent and opposing effects on osteoblast activity. Markers of osteoblast maturation and activity were assessed by semi-quantitative RT-PCR for interactions between OSM signaling and RCCS. Representative images at 48 h are shown (A), when the largest effects were found. For markers affected by OSM or RCCS, the results of densitometry are shown as scatter plots with mean and SEM (B-D). OSM supplementation induced collagen α1(I) (B) and osteocalcin (C) transcription, but had no significant effect on sclerostin transcription (D). RCCS inhibited the transcription of collagen α1(I) and osteocalcin, while increasing transcription of sclerostin. No significant interaction between the factors was detected by ANOVA.

To determine if the interdependence of osteoblast OSM signaling and RCCS extended to their effects on osteoblast maturation and activity, we analyzed samples collected over the course of a week in these conditions by semi-quantitative RT-PCR for several markers of osteoblast differentiation and osteoid production (Fig 5A). Significant effects were found for collagen α1(I) (Fig 5B), osteocalcin (Fig 5C), and sclerostin (Fig 5D). Independently, OSM treatment and RCCS acted on collagen α1(I) and osteocalcin, as would be expected for these components of osteoid and markers of middle and late osteoblast maturation. Both had mRNA levels increased by OSM and decreased by RCCS. Sclerostin expression was increased by RCCS, suggesting an increase in the osteocyte character of these cell cultures and consistent with evidence that microgravity inhibits osteoblast differentiation. No significant interaction between these factors was detected by ANOVA, which suggests that simulated microgravity does not alter the effect of OSM on osteoblast activity.

### Limitations associated with using this RCCS and Cytopore cell culture model to measure the effects of microgravity on osteoid formation and mineralization

**Fig 6:**
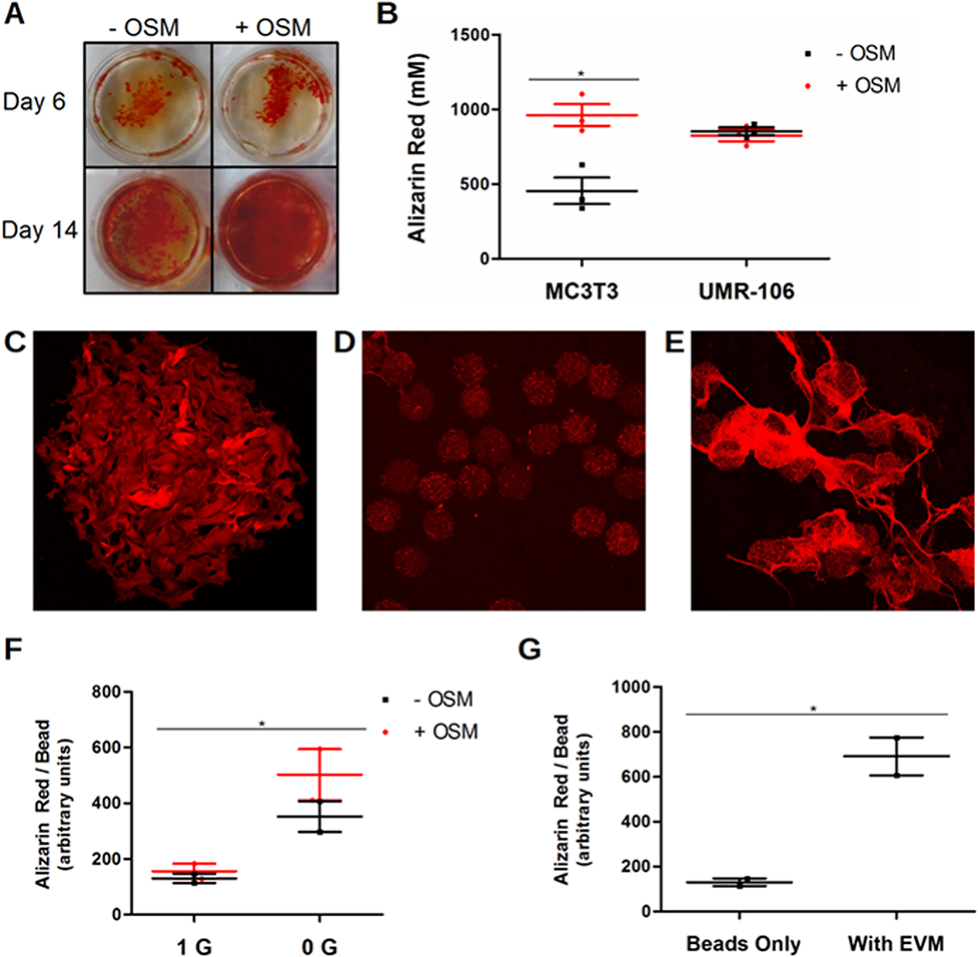
Limitations associated with using this RCCS and Cytopore cell culture model to measure the effects of microgravity on osteoid formation and mineralization. (A) Alizarin red staining of osteoid mineralization after culture of MC3T3-E1 cells for 6 or 14 days in mineralizing culture medium. OSM increases production of mineralizing osteoid. (B) Quantification of the differential effect of OSM on mineralization after 14 d in monolayer culture for the MC3T3-E1 mouse pre-osteoblast and UMR-106 rat osteosarcoma cell lines. (C) Confocal micrograph (400x) of fluorescent alizarin red bound to mineralization in MC3T3-E1 culture on Cytopore microcarriers for 14 d. (D) Representative confocal micrograph (50x) of MC3T3-E1 cultured 14 d on Cytopore with mineralization stained for quantification by densitometry. (E) Confocal micrograph (50x) of Cytopore MC3T3-E1 culture in control gravity conditions showing mineralized osteoid. (F) Densitometry showed greater mineralization in RCCS cultures. (G) Mineralized osteoid accounts for the majority of mineralization under control gravity conditions.

The effect of OSM on the production of mineralized osteoid was also investigated. The organic dye alizarin red specifically stains mineralization in osteoid (Fig 6A), allowing visualization of the differences in osteoid production in cell culture. The dye can also be extracted and quantified spectrophotometrically [62]. This aided in the choice of the MC3T3-E1 cell line to study OSM's effect in osteoblasts. The MC3T3-E1 cell line was chosen for these experiments in part because of the clear effect OSM has on culture mineralization, whereas the UMR-106 rat osteosarcoma cell line, for example, shows no effect (Fig 6B).

A different approach to mineral quantification was used for MC3T3-E1 cells cultured on Cytopore microcarriers when the extraction technique proved insufficiently sensitive. Because alizarin red fluoresces when excited by light at 530–560 nm, staining could be visualized with laser confocal microscopy (Fig 6C). It proved possible to quantify the staining by densitometry, and thus mineralization, on samples of microcarriers (Fig 6D and 6F). While reproducible, the results from this technique showed substantially higher mineralization in RCCS (Fig 6F), not at all consistent with the known effects of spaceflight [2,3]. During staining, large quantities of what proved to be mineralizing material were sometimes noted (Fig 6E). This material, presumed to be osteoid, was only found in normal gravity control cultures, never in samples from the RCCS. It appears the microcarriers must be stationary for the osteoblasts to produce mineralized osteoid. In the control gravity samples, the mineralized osteoid was generally disrupted and lost during staining and washing, so that most of it could not be quantified. When the remnant mineralized osteoid was quantified, however, it substantially exceeded the mineral found on only the microcarriers (beads) from the same samples (Fig 6F). If mineralized osteoid could have been preserved in the final staining, it is likely that total mineralization would have been much higher in control gravity samples compared to RCCS conditions. From our observations, we conclude that the RCCS model of microgravity, used with cotton cellulose-based Cytopore microcarrier beads, is unable to reproduce the patterns in mineral formation seen in actual spaceflight data. Other types of beads may need to be considered for future studies such as collagen-based, or hydroxyapatite-activated bacterially spun cellulose-based microcarriers or scaffolds [63].

## Discussion

Understanding how microgravity disrupts osteoblast function is important for human space exploration, as well as for patients on earth experiencing extended bed rest or disuse osteoporosis. There are many uncertainties in the action of inflammatory signaling in the regulation of bone remodeling. The results presented here connect the questions of microgravity's action on MC3T3-E1 osteoblastic cells and the function of the GP130 cytokines. In particular, they support the hypothesis that microgravity and ionizing radiation alter the function of OSM signaling in osteoblasts. The pattern seen in the disruption of the effects of OSM is consistent with the increase in osteolysis and decrease in osteogenesis seen in astronauts.

The most prominent effect of our model of simulated microgravity on osteoblast OSM signaling was on GP130 signaling itself, particularly the secretion of IL-6. We described here an increase in IL-6 secretion by RCCS and the synergistic increase by the combination of RCCS and OSM supplementation. The increase in secretion is closely paralleled by an increase in IL-6 mRNA. The degree of increase in IL-6 secretion cannot be explained by a simple combination of the individual effects of RCCS and OSM. This is clear evidence that one affects the other. One possible explanation for this is that RCCS interferes with feedback inhibition mechanisms regulating OSM signal transduction or IL-6 secretion. Inhibition of OSM and IL-6 signaling by suppressor of cytokine signaling (SOCS) protein is an example of an important regulatory pathway that is known to be involved in skeletal health and could be inhibited in microgravity [64,65]. As IL-6 acts in bone to increase the recruitment of osteoclasts [42,43], this increase in IL-6 secretion can reasonably be expected to contribute to osteolysis in microgravity. This is particularly significant considered alongside the data collected from astronauts suggesting that GP130 signaling is generally altered in spaceflight and IL-6 levels increased in particular [31–34]. While not dependent on RCCS, the observed increase in OSMRß and IL-6Rα mRNA is consistent with a positive feedback mechanism for OSM and IL-6 signaling that may be important in understanding the general action of these cytokines on osteoblasts.

OSM's regulation of osteoblast IL-6 secretion is further complicated in a spaceflight model that includes microgravity and radiation. Our results showed that radiation alone or in combination with RCCS increased IL-6 secretion, as did OSM treatment. Radiation in combination with OSM, however, diminished the OSM-induced secretion by as much as half. While radiation alone, as a single challenge, has historically been thought to cause a persistent reduction of osteoblast activity, more recent studies have uncovered a more complex response that may be driven by inflammatory-mediated processes versus cell death [66]. For example exposures to less than 2 Gy promote osteoblast differentiation and osteoid production [67–69]. At doses above 2 Gy, bone formation has been observed to both decrease [70–73] and increase at certain time points [73,74]. Yumoto and colleagues have also observed that the combination of irradiation with unloading may also determine the effect of ionizing radiation on osteoblast function [75], as we observed here. Applying these observations to our data, it is reasonable that the combined effect of OSM and radiation differs from their individual effects. It is also noteworthy that factors other than dose can affect osteoblast response to radiation. Linear energy transfer (LET) may be an important factor [36,76,77], which should be considered in evaluating our model that only uses low-LET photons. The radiation from solar particle events (SPE) and galactic cosmic rays (GCR) in spaceflight has a large high-LET component [7]. While it is thought that high-LET radiation is generally more toxic than low-LET, it has other effects such as causing changes in epigenetic expression and differential production of miRNA levels [78,79]. As noted, the few reports using high-LET radiation (e.g., Fe^56^ ions) on bone formation and physiology show varied responses often related to dose and timing after exposure [68,75,80,81]. Therefore, the effect of high-LET radiation on bone is still not very well understood and further studies are needed to more accurately assess GCR effects on bone. As we have shown, many factors must be considered in any attempt to understand the osteoblast response to radiation.

In summary, OSM treatment, modeled microgravity using the RCCS, and radiation all independently increase the secretion of IL-6. OSM has the largest and most immediate effect. RCCS and radiation both a have more modest effect on IL-6 secretion that presents more slowly than the effect from high levels of OSM supplementation. The difference in timing may only be apparent due to the inability of our assays to detect the comparatively smaller early effects, or it may indicate that OSM acts more directly on IL-6 transcription than RCCS or radiation. These explanations are not mutually exclusive.

We have also described a dependence on RCCS for the action of OSM on osteoblast-mediated osteoclast recruitment. In control gravity conditions, active OSM signaling had no effect on the RANKL: osteoprotegerin ratio. In RCCS, OSM signaling increased osteoprotegerin levels. On its own, this is further evidence that microgravity alters the action of OSM signaling in the osteoblast, which supports our central hypothesis. It also demonstrates that RCCS depends on the presence or absence of other factors for its effects. By implication, the effect of microgravity on astronauts may depend on factors that vary between individuals, such as baseline inflammatory cytokine levels. It cannot be determined from these experiments how this feature of OSM signaling in RCCS, which would oppose increased osteoclastogenesis, balances with the increase in IL-6 secretion and its support of osteoclast recruitment. Although these experiments did not support microgravity acting through OSM signaling to affect osteoblast activity and maturation, it is worth noting that they focused on the osteoblast-committed MC3T3-E1 cell line. OSM is known to act throughout the osteoblast lineage from uncommitted precursors to osteocytes [51,82,83]. One can imagine that microgravity would alter the effect of OSM signaling at other points in the osteoblast lineage. Our results imply that inflammatory signaling pathways must be considered in understanding the action of microgravity on osteoclast activity.

It is worthwhile to consider the inability of this system to model changes in osteoid production and mineralization in microgravity. Mineralized osteoid could not form in RCCS, most likely due to the microcarriers' constant motion and shear forces. The ability of the osteoblasts to move beyond the microcarrier beads appears to have been critical for the organization and amount of osteoid they produced. That ability to move and interact in a larger space may also have been critical in successfully modeling the decrease in osteoblast activity seen in microgravity. Also Cytopore beads are manufactured from a plant source using cotton as a base for the cellulose beads and may not promote accurate model of mineralization. Other types of microcarriers can also be used that may help model bone environment more accurately such as collagen-based microcarriers or more bio-compatible bacterially-spun cellulose scaffolds [63].

**Fig 7:**
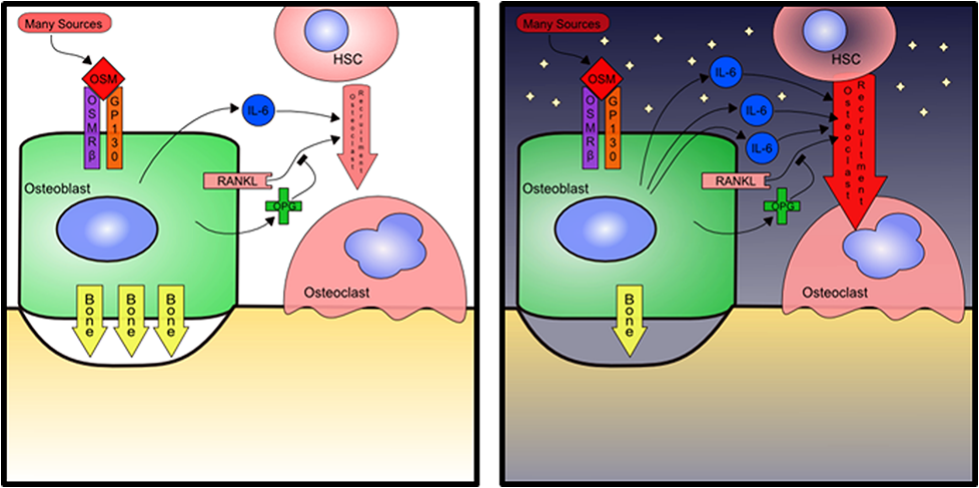
Summary model of microgravity and OSM promoting net bone loss. Under normal (1G) gravity conditions (left), OSM promotes osteoblast activity and stimulates osteoid production. OSM also promotes the expression of osteoblast produced IL-6 and RANKL to promote osteoclast differentiation and activity, leading to bone resorption. This activity is regulated by the production of OPG, which will inhibit RANKL-mediated effects on osteoclasts. Under microgravity conditions (right), osteoblast activity is inhibited, while the production of osteoclast activating IL-6 by the osteoblasts is increased when stimulated by OSM. Overall, osteoid formation by osteoblasts is reduced and osteoclast activity increases under microgravity, leading to net bone loss.

Overall, microgravity and radiation act independently of OSM to increase osteoblast-mediated production of RANKL and IL-6 and suppression of osteoprotegerin, where these effects may cause increased osteoclast activity. Meanwhile, microgravity inhibits the activity of MC3T3-E1 osteoblasts, which when combined with effects on osteoclasts through signaling may both promote osteolysis and inhibit replacement with new bone. Additionally, microgravity synergizes with OSM to increase IL-6 production further without any balancing effect on osteoblast activation, so that the overall effect of OSM on osteoblasts in microgravity may promote bone loss. The effects of OSM may therefore contribute to the uncoupling of bone formation and resorption that occurs during spaceflight. This interpretation of our results is summarized graphically in Fig 7. The role that osteoblasts play in bone is complex, where many effects depend on the spatial and temporal interaction of many different cells. It is difficult to predict which features of that complexity will be important for a given question, in bone or anywhere in biology. The most valuable contribution of this work may be pointing to GP130 signaling as a feature that must be included in a complete picture of osteoblast activity in space.
